# Hydrophobia—Rabies—Prophylaxis

**Published:** 1898-05

**Authors:** 


### HYDROPHOBIA—RABIES—PROPHYLAXIS.

G. Archie Stockwell,[1] in a lengthy article on this subject, concludes with a few simple axioms, which, if heeded, will, in the majority of cases, allay fear and quell the senseless epidemics of rabiphobia that threaten from time to time:

Rabid dogs do not fear, but court water.

Rabies can never arise spontaneously either in animals or man; the absence of recent wounds upon and the good health of the creature inflicting the bite are alike evidence of the non-specific character of the disease.

Wounds inflicted by the teeth of non-carnivorous creatures are never rabic, though a blood-poisoning may be induced; man can not communicate the disease to other human beings or to animals.

The saliva of truly rabid carnivora alone is capable of inducing rabies.

Excessive saliva in any creature, more especially dogs, is always induced by any affection of the mouth or throat; it is the universal sequel of toothache, of inability to swallow from any cause, of paralysis, of throat-abscess. Paralysis independent of rabic condition is a sequel to numerous local brain and nerve maladies. Twitching of eyes, of eyelids, of lips and muscles; lolling tongue, in-drawn tail, up-drawn flanks, drooping head, slinking gait—one and all should be included in the same category.

Safety lies in preserving life of the suspected canine and sequestrating for observation; by premature death the only evidence whereby rabies can definitely and with certainty be established is destroyed. Muzzle the creature and examine for recent wounds; if none is exhibited, fear is idle.

If a dog evinces a good appetite, and if it partakes of food freely on and after the fourth day, it is not rabid; if it dies without paralysis, it is not rabid; if it succumbs to paralysis, the chances are still a thousand to one that its death was due to some natural cause; if it falls into convulsions and froths at the mouth, it is an epileptic and an object of commiseration; ninety-nine of every hundred dogs pronounced "mad" are epileptics or choreics, and dogs with "fits" are positively never rabid; if, after ten days' confinement, the suspected animal evinces a cheerful disposition, there is no danger whatever and it should be released.

When death supervenes, critical examination of the body—the brain, viscera, etc.—should be made by a careful, competent pathologist, to determine whether there is a cause for death *aside* from rabies.

Members of the human race suffering from rabies do not howl, whine, bark, or exhibit other canine characteristics or peculiarities any more than do cats, fowls, sheep, cattle, etc.;

---

1Med. Age, Oct. 10-25, 1897; Dom. Med. Mo., Dec., 1897.

such phenomena are conclusive evidence the malady is—*hysterical!*

Intolerance of light and of moving air, fear of water and bright substances, and spasm of muscles of the throat and neck, far from constituting specific evidence, are common in diphtheria, croup, goitre, sore throat, lock-jaw, pregnancy, hysteria, ear-abscess, hay fever and poisoning by certain mineral and vegetable substances; they may arise from the use of narcotics, sedatives and alcoholics; from cancerous affections; certain maladies of the internal ear, or the eye, as in glaucoma; diseases peculiar to sex, and a multitude of brain and nerve maladies. In no instance would these symptoms excite special comment or alarm were not the dog linked therewith as a factor.

Error of judgment is the almost inevitable result of accepting mere negation for their antithesis; careful examination of the evidence laid down for the distinction of rabies reveals little aside from mere negations; until death ensues, the weight of evidence is always negative; when dissolution follows, it is very far from being presumptive of the positive.

Since less than three per cent of infected canines ever contract the malady, the danger to man is several hundred times less than from scarlatina, measles, quinsy, ague and a host of simple maladies. As Charles Bell Taylor remarks, "It is less than one in a million—less than being kicked to death by a horse," to which may be added: it is less than the gallows. Finally, after examining as thoroughly as possible the history of every case of rabies reported as occurring in North America during fifty years (and they number about one thousand), Stockwell has been unable to find even one that was not open to the gravest suspicion as to error.

Law[1] details a supposed case of rabies, diagnosed as such by every physician in Ithaca, N. Y., which, under a dose of castor-oil and a tonic, recovered within a week.

Smith, of Virginia,[2] states that rabies does not exist in his State, and that it will take better evidence than has yet been produced to convince him that it exists anywhere.

Dulles[3] has carefully studied rabies, or hydrophobia, from every standpoint for sixteen years. During two years he critically examined forty cases. He insists that rabies is not a specific disease, and that the term "hydrophobia" should, if employed at all, be used in the same sense as "convulsions," without prejudice as to the cause of the phenomena. There can be no doubt that death with a peculiar train of symptoms has often followed dog-bite, but these symptoms and the usual fatal issue are nowise peculiar to injuries inflicted by animals believed to be rabid. There are many cases in which the belief of madness of the dog rests wholly upon the

1 Proc. U. S. Vet. Med. Assoc., 1897.

2 *Ibid.*

3 Proc. Med. Soc. of Pa., 1897; Med. Age, July 10, 1897.

result in the person bitten, and it is not scientific to assume that this belief is more than a belief, and no person fit to discuss the question would treat it as a demonstration. Further, the symptoms appear in a great many diseases, and persons bitten by dogs enjoy no immunity from these diseases. Besides this, there are many cases in which persons have died of hydrophobia while the dogs have survived. There are other cases in which the symptoms of hydrophobia have appeared after various traumatism, and such a traumatism as is caused by a dog-bite may (without assuming the presence of a specific virus) lead to nervous symptons and death. A curious factor in this connection is that dog-bites cause so many deaths in what may be termed the private walks of life, and practically none among men who are constantly handling large numbers of dogs, including persons who habitually take up stray dogs on the streets, such as dog-catchers, policemen (as in London) and attendants of such institutions as Homes for Lost and Straying Dogs. In the home in London over 200,000 canines have been handled without a case of rabies or hydrophobia; among keepers of kennels it is the same, though they may have been bitten several times, many of the wounds inflicted by dogs believed by their owners to be rabid. It is a remarkable fact, also, that, though there has been such a fever of excitement about rabies in Paris in recent years, there have been no deaths among dog attendants and "pound-keepers." If the bites received by the foregoing people could be counted, the customary ratio of deaths to bites would have to be enormously modified. The oft-quoted guess of John Hunter, of one to twenty-four, would probably require to be exchanged for one to seventy-five or perhaps one to one hundred.

[Fabre says the ratio is less than one to six hundred. ED.] —*Monthly Cyclopedia of Practical Medicine.*

